# Exploring functionality of the reverse β-oxidation pathway in *Corynebacterium glutamicum* for production of adipic acid

**DOI:** 10.1186/s12934-021-01647-7

**Published:** 2021-08-04

**Authors:** Jae Ho Shin, Aaron John Christian Andersen, Puck Achterberg, Lisbeth Olsson

**Affiliations:** 1 Department of Biology and Biological Engineering, Division of Industrial Biotechnology, Chalmers University of Technology, Gothenburg, Sweden; 2 Department of Biotechnology and Biomedicine, Technical University of Denmark, Lyngby, Denmark; 3 Present Address: Department of Biotechnology, Delft University of Technology, Delft, The Netherlands

## Abstract

**Background:**

Adipic acid, a six-carbon platform chemical mainly used in nylon production, can be produced via reverse β-oxidation in microbial systems. The advantages posed by *Corynebacterium glutamicum* as a model cell factory for implementing the pathway include: (1) availability of genetic tools, (2) excretion of succinate and acetate when the TCA cycle becomes overflown, (3) initiation of biosynthesis with succinyl-CoA and acetyl-CoA, and (4) established succinic acid production. Here, we implemented the reverse β-oxidation pathway in *C. glutamicum* and assessed its functionality for adipic acid biosynthesis.

**Results:**

To obtain a non-decarboxylative condensation product of acetyl-CoA and succinyl-CoA, and to subsequently remove CoA from the condensation product, we introduced heterologous 3-oxoadipyl-CoA thiolase and acyl-CoA thioesterase into *C. glutamicum*. No 3-oxoadipic acid could be detected in the cultivation broth, possibly due to its endogenous catabolism. To successfully biosynthesize and secrete 3-hydroxyadipic acid, 3-hydroxyadipyl-CoA dehydrogenase was introduced. Addition of 2,3-dehydroadipyl-CoA hydratase led to biosynthesis and excretion of *trans*-2-hexenedioic acid. Finally, trans-2-enoyl-CoA reductase was inserted to yield 37 µg/L of adipic acid.

**Conclusions:**

In the present study, we engineered the reverse β-oxidation pathway in *C. glutamicum* and assessed its potential for producing adipic acid from glucose as starting material. The presence of adipic acid, albeit small amount, in the cultivation broth indicated that the synthetic genes were expressed and functional. Moreover, 2,3-dehydroadipyl-CoA hydratase and β-ketoadipyl-CoA thiolase were determined as potential target for further improvement of the pathway.

**Supplementary Information:**

The online version contains supplementary material available at 10.1186/s12934-021-01647-7.

## Introduction

The detrimental impact of petrochemical-based manufacturing on the environment has increased demand for biomass-derived chemicals, fuels, and consumer products [1, 2]. Biomass-based derived compounds now include fuels [3], monomers [4], polymers [5], and pharmaceuticals [2], with a few products already exerting a significant impact in the chemical industry [6, 7]. Global ongoing efforts are focused on expanding the portfolio, as well as increasing titers and yield.

Adipic acid, an aliphatic dicarboxylic acid, has gained wide attention from the metabolic engineering community as a six-carbon platform chemical for more than two decades [8]. Adipic acid is used as a building block for nylon-6,6, as well as other fibers, polyesters, and resins [9]. Its industrial production volume amounts to nearly 3 million US tons and is achieved via chemical oxidation of petroleum-derived KA oil (cyclohexanone and cyclohexanol) [10]. The petroleum-dependent nature of adipic acid production, has intensified the search for sustainable adipic acid precursors, derivatives, and analogues [11–13].

While bio-based production of adipic acid had been hampered by lack of efficient pathways, both synthetic [14–20] and naturally occurring pathways [21] have been characterized. *Thermobifida fusca* [21], as well as engineered *Escherichia coli* [14–20, 22], *Saccharomyces cerevisiae* [10], and *Pseudomonas putida* [23] can generate adipic acid from bio-based carbon sources, such as glucose or glycerol. As reviewed previously [9, 24–27], successful biosynthesis of adipic acid has often exploited the reverse β-oxidation pathway. This pathway is a synthetic extension of the tricarboxylic acid (TCA) cycle, has favorable thermodynamics (− 78.4 kJ/mol) [19], and leads to biosynthesis of coenzyme A (CoA)-activated intermediates via condensation of TCA metabolites, succinyl-CoA and acetyl-CoA. Whereas glycerol is an especially good carbon source for the production of adipic acid *via* reverse β-oxidation [15], the same has not been demonstrated for glucose. Reverse β-oxidation genes from combinatorial genetic sources have been applied in *E. coli* due to its relatively easy engineering [14, 16–19], whereas native adipic acid metabolic pathways have been identified in *T. fusca* [21]. Since the demonstration of adipic acid production from glucose in *T. fusca*, reverse β-oxidation genes from this bacterium have been applied in *E. coli* to attain high-titer adipic acid production from glycerol [15]. To further improve adipic acid titers from monosaccharides and ultimately lignocellulosic materials, new strategies are required.

*Corynebacterium glutamicum* is an established industrial workhorse for producing amino acids, such as L-glutamate [28] and L-lysine [29, 30]. New genetic engineering and bioinformatics tools have expanded the chemical product portfolio of *C. glutamicum* to include L-valine [31], pinene [32], diaminopentane [29], γ-aminobutyrate [33], 5-amiovaleric acid [34, 35], glutaric acid [36], alcohols [37], resveratrol [38], and muconic acid [39, 40]. Given that, unlike *E. coli*, *C. glutamicum* does not naturally consume glycerol [41], the majority of aforementioned studies on *C. glutamicum* have focused on simple sugars as carbon sources. Notably, bio-based production of 1,5-diaminopentane [42], 5-aminovaleric acid, and glutaric acid [43] has achieved higher titers in *C. glutamicum* than in *E*. *coli* [34–36, 44], likely due to higher metabolic flux toward amino acids in the former species [29]. Furthermore, engineered *C. glutamicum* can produce industrial titers of succinic acid (up to 146 g/L) [45], greatly surpassing other organisms [46]. Because the reverse β-oxidation pathway begins with TCA cycle intermediates and extensive amounts of succinic acid can be generated in *C. glutamicum* [47], introduction of adipic acid biosynthesis pathways in this organism has been proposed here in this study.

**Fig. 1:**
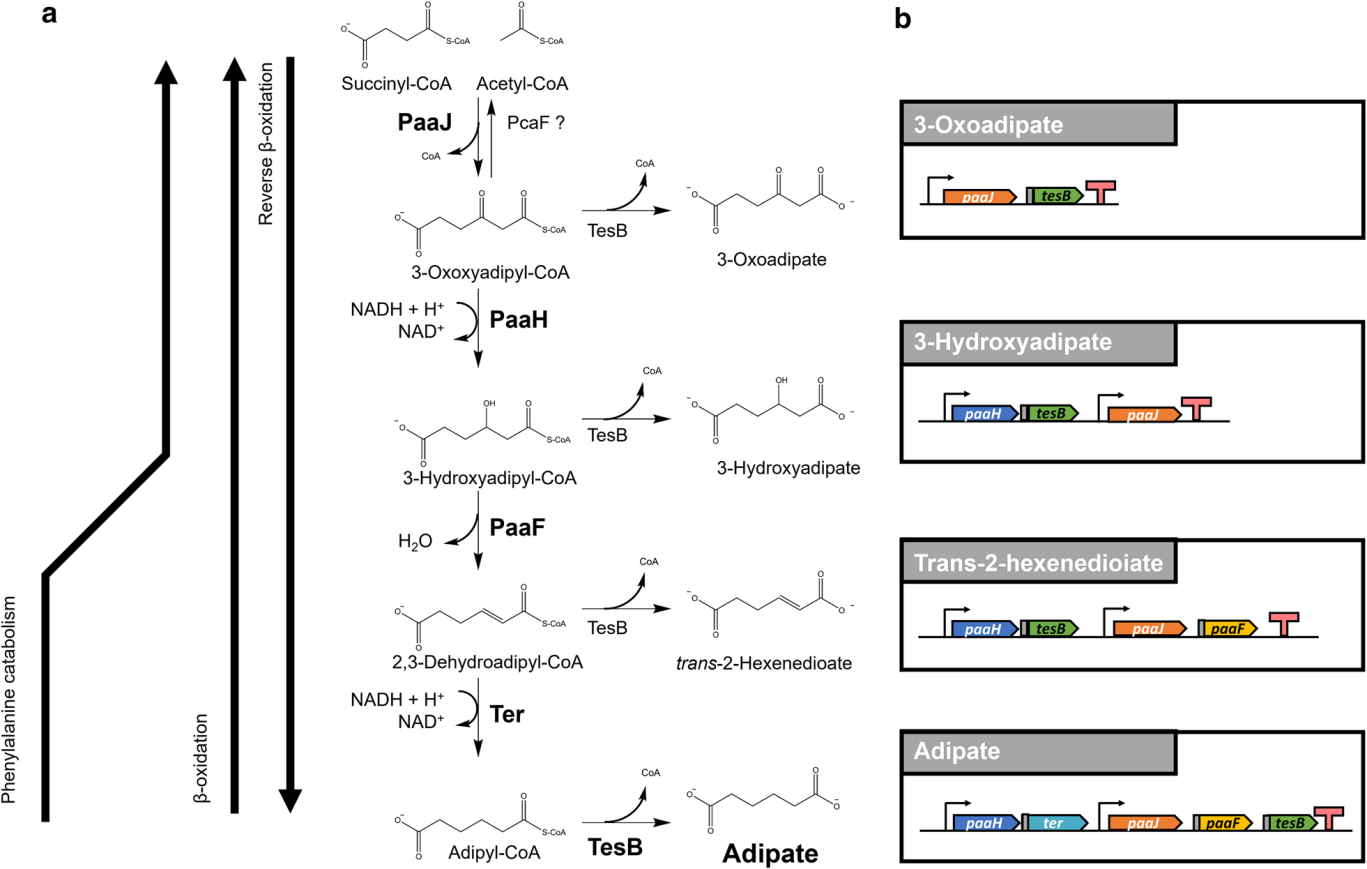
Reverse β-oxidation pathway for adipic acid biosynthesis from TCA metabolites.** a** Synthetic pathway designed to enable adipic acid biosynthesis from succinyl-CoA and acetyl-CoA. Five enzymatic steps, including condensation, reduction, dehydration, second reduction, and hydrolysis of a thioester bond, are carried out by *paaJ, paaH, paaF*, *ter*, and *tesB* gene products encoding the *E. coli* PaaJ thiolase, PaaH 3-hydroxyacyl-CoA dehydrogenase, PaaF 2,3-dehydroadipyl-CoA hydratase, Ter *trans*-2-enoyl-CoA reductase, and TesB thioesterase. PcaF corresponds to a putative acetyl-CoA:acetyltransferase. **b** Vector design to investigate pathway intermediate compounds

In the present study, we explored the generation of adipic acid using *C. glutamicum* as proof-of-concept cell factory. While adipic acid production from glucose has been attempted mostly in *E. coli* [14–20], development of other organisms may lead to potential improvements. To this end, we designed a synthetic pathway consisting of five heterologous genes (Fig. 1) and implemented reverse β-oxidation in *C. glutamicum* using glucose as carbon source. According to the designed scheme, *E. coli* 3-oxoadipyl-CoA thiolase (PaaJ; E.C. 2.3.1.174) [14, 16, 19] catalyzes the condensation of acetyl-CoA and succinyl-CoA to form a six-carbon backbone. Next, the resulting 3-oxoadipyl-CoA is reduced by *E. coli* 3-hydroxyacyl-CoA dehydrogenase (PaaH; E.C. 1.1.1.157), and then dehydrated by *E. coli* 2,3-dehydroadipyl-CoA hydratase (PaaF; E.C. 4.2.1.17) [16, 19]. Subsequently, reduction by *trans*-2-enoyl-CoA reductase (Ter; E.C. 1.3.1.44) from *Treponema denticola* [48] is followed by release of CoA via thioesterase (TesB) from *Acinetobacter baylyi* [49] to finally yield adipic acid (Fig. 1).

## Results

### Assessment of non-decarboxylative Claisen condensation of acetyl-CoA and succinyl-CoA in *C. glutamicum* by overexpressing *paaJ* from *E. coli*

The design of synthetic pathway (Fig. 1) is motived by the similarities found in the bacterial phenylalanine catabolism pathways. Catabolism of phenylalanine in *E. coli* and styrene in *Pseudomonas* proceed *via* phenylacetate degradation [50–52]. In the last steps of phenylacetate catabolism, 2,3-dehydroadipyl-CoA is hydrated to 3-hydroxyadipyl-CoA, which is then oxidized to yield 3-oxoadipyl-CoA. Subsequently, succinyl-CoA and acetyl-CoA formed by thiolytic cleavage of 3-oxoadipyl-CoA are assimilated in the TCA cycle. Overexpression of *E. coli paaF*, *paaH*, and *paaJ* genes involved in these steps previously has demonstrated not only the reversibility of the individual enzymes, but also that of the pathway’s direction [16, 19]. Additionally, CoA-released byproducts of the abovementioned pathway, including 3-oxoadipate, 3-hydroxyadipate, and *trans*-2-hexenedioate, have been observed in the cultivation broth of *E. coli* overexpressing *paaJ*, *paaH*, and *paaF* [19]. While reverse β-oxidation may function through multiple cycles [53], PaaJ was shown to stop after the first cycle when overexpressed in *E. coli*, thus preventing the formation of C8 or C10 compounds [16]. Accordingly, here, *E. coli* PaaJ was chosen as the first reaction step (Fig. 1).

**Fig. 2:**
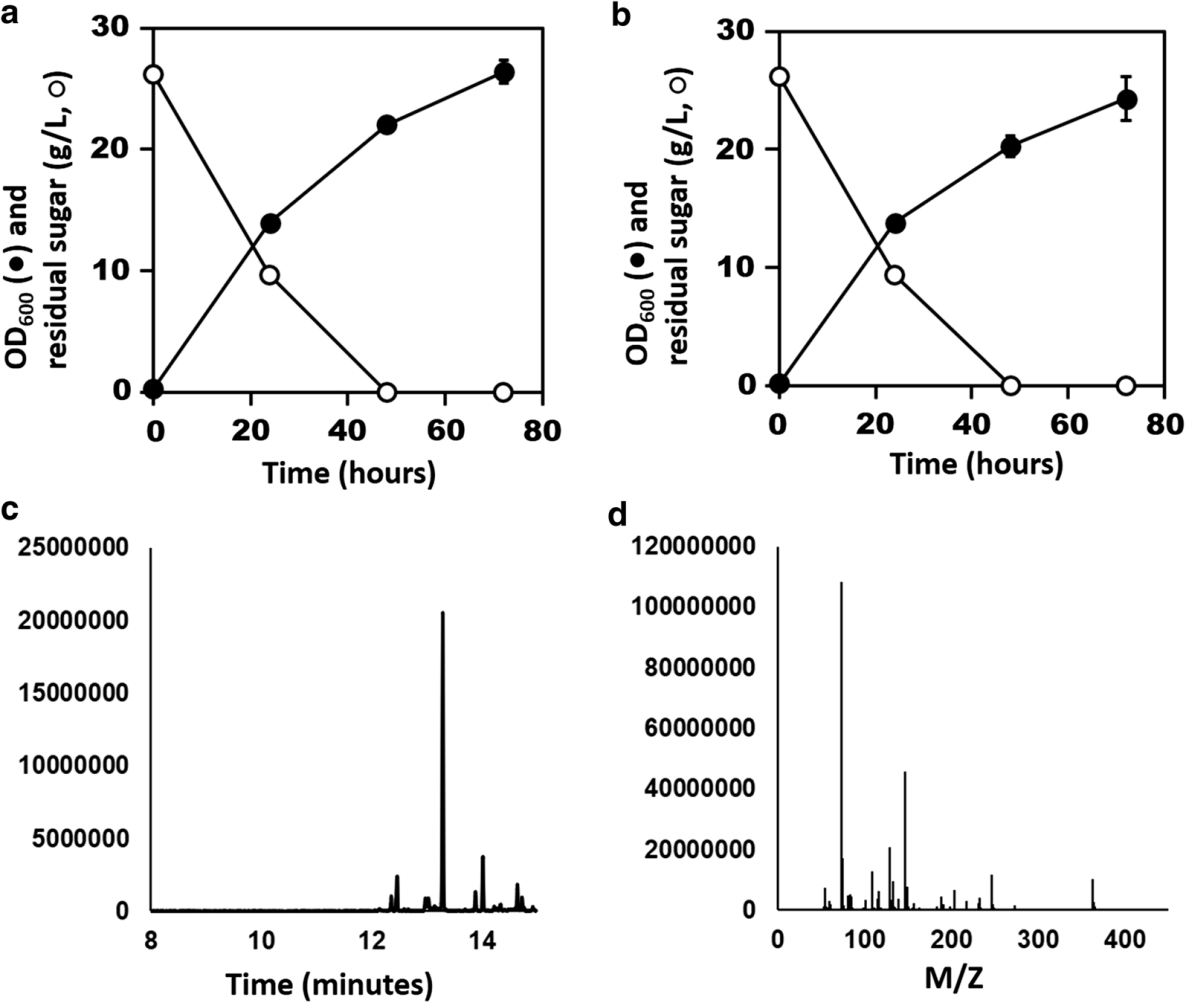
Biosynthesis of adipic acid pathway intermediates from glucose. Shake-flask cultivation profile of *C. glutamicum* harboring (**a**) pZ8-paaJ-tesB and (**b**) pZ8-paaH-tesB-paaJ. OD_600_ (filled circles), residual sugar (empty circles). N = 3; error bars = standard deviation. **c** Representative GC/MS ion (*m/z* = 363) extracted chromatogram of MSTFA-derivatized cultivation broth from *C*. *glutamicum* transformed with pZ8-paaH-tesB-paaJ. The peak at retention time 13.3 min corresponds to MSTFA-derivatized 3-hydroxyadipic acid. (**d**) *m/z* fragmentation pattern of the peak corresponding to MSTFA-derivatized 3-hydroxyadipic acid from (**c**)

To assess whether non-decarboxylative Claisen condensation of succinyl-CoA and acetyl-CoA into 3-oxoadipyl-CoA occurred in *C. glutamicum*, the *E. coli paaJ* gene (KEGG ID: JW1392) was cloned into pZ8-P*tac* (Fig. 1; Additional file 1: Table S1, Additional file 2: Table S2), an *E*. *coli*-*C*. *glutamicum* shuttle vector [54], and the CoA-released byproduct of 3-oxoadipyl-CoA was measured. TesB from *A*. *baylyi* is thought to have a broad substrate specificity and is known to release CoA from 3-oxoadipyl-CoA [19]. Similarly, *E. coli* thioesterase, which shares 43 % identity with *A. baylyi* TesB, has been shown to release adipic acid from adipyl-CoA at its endogenous basal expression level [16]. Thus, *tesB* from *A. baylyi* and *paaJ* from *E. coli* were organized in an operon behind the native *tac* promoter in the pZ8-paaJ-tesB vector (Fig. 1; Additional file 1: Table S1**)**. *C*. *glutamicum* was transformed with pZ8-paaJ-tesB and its growth and capability to synthesize 3-oxoadipic acid by flask-batch cultivation were assessed (Fig. 2a). *C. glutamicum* harboring pZ8-paaJ-tesB reached OD_600_ of 26.4 after 72 h. The metabolites excreted in the cultivation broth were derivatized with *O*-methylhydroxylamine (MeOX), and *N-*methyl-*N*-(trimethylsilyl)trifluoroacetamide (MSTFA), and analyzed by GC/MS. As 3-oxoadipic acid could not be detected (Additional file 3: Fig. S1), we speculated that it was metabolized and, instead, decided to measure the next byproduct of the pathway.

### Demonstration of 3-hydroxyadipate biosynthesis from engineered *C. glutamicum*

To biosynthesize and detect 3-hydroxyadipate (Fig. 1), we cloned *paaJ*, *tesB*, and a codon-optimized version of *E*. *coli paaH* (KEGG ID: JW1390) in the pZ8-P*tac* backbone. 3-hydroxyadipate has previously been detected in recombinant *E*. *coli* [19]. To ensure sufficient expression of *paaJ*, *tesB*, and *paaH* from a vector-based operon in *C. glutamicum* [35], the new pZ8-paaH-tesB-paaJ plasmid included an extra *tac* promoter [55]. The latter was added to the native *tac* promoter upstream of *paaJ* to ensure expression of *paaJ* and *paaH*. Owing to possible activity of endogenous *C. glutamicum* thioesterases, the *tesB* gene accompanied by an additional ribosome-binding site (RBS) was placed downstream of *paaH*. *C. glutamicum* transformed with pZ8-paaH-tesB-paaJ reached OD_600_ of 24.3 after 72 h of flask-batch cultivation (Fig. 2b). MSTFA derivatization of the cultivation broth followed by GC/MS revealed the presence of 3-hydroxyadipic acid among excreted metabolites (Fig. 2c, d). The retention time of trimethylsilyl-3-hydroxyadipic acid was 13.3 min under our experimental conditions. The *m/z* pattern of the compound matched that of the MSTFA-reacted 3-hydroxyadipic acid standard (Additional file 4: Fig. S2). These findings demonstrated successful heterologous overexpression of *paaJ*, *paaH*, and *tesB*, resulting in production and excretion of 3-hydroxyadipate from engineered *C*. *glutamicum*. Moreover, the presence of 3-hydroxyadipic acid in the cultivation broth indirectly confirmed the correct functioning of PaaJ and condensation of acetyl-CoA and succinyl-CoA in engineered *C. glutamicum*. Thus, overexpression of PaaJ in *C*. *glutamicum* allows manipulation of the thermodynamic equilibrium between acetyl-CoA, succinyl-CoA, and 3-oxoadipyl-CoA, in spite of endogenous β-ketoadipyl CoA thiolase (PcaF), which is thought to promote the catabolic direction of the pathway [56].

### Demonstration of *trans*-2-hexenedioic acid from engineered *C. glutamicum*

For *trans*-2-hexenedioic acid biosynthesis in *C. glutamicum*, PaaF (KEGG ID: b1393) from *E. coli* [50] was added to pZ8-paaH-tesB-paaJ. The resulting vector was named pZ8-paaH-tesB-paaJF and transformed into *C. glutamicum*. PaaF from *E*. *coli* catalyzes the dehydration of 3-hydroxyadipyl-CoA [16, 19], forming 2,3-dehydroadipyl-CoA. The CoA-released form of the latter compound, *trans*-2-hexenedioic acid, has been detected from an engineered *E. coli* strain [19].

**Fig. 3:**
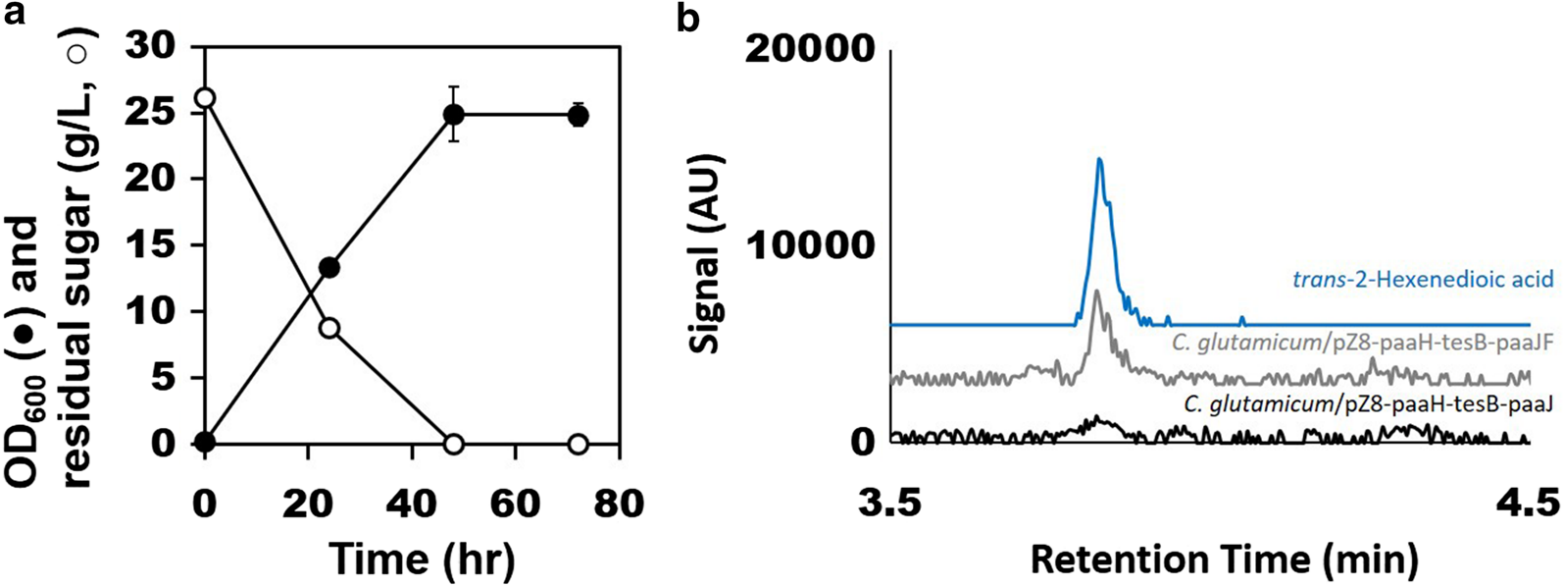
Shake-flask cultivation profile of *C. glutamicum* harboring pZ8-paaH-tesB-paaJF. **a** Cultivation profile including OD_600_ (filled circle) and residual sugar (empty circle). N = 3; error bars = standard deviation. **b** Ion (*m/z* = 143.03498) extracted chromatogram obtained by LC/MS of *trans*-2-hexenedioic acid (blue), cultivation broth of *C. glutamicum* harboring pZ8-paaH-tesB-paaJF (grey), and a control vector (black)

The engineered *C. glutamicum* strain expressing the four genes reached OD_600_ of 24.8 after 72 h shake-flask cultivation (Fig. 3a). Concentrating the cultivation broth 5-fold using solid-phase extraction followed by LC/MS revealed presence of *trans*-2-hexenedioic acid (Fig. 3b). *C. glutamicum* strain harboring pZ8-paaH-tesB-paaJ did not produce detectable amount of *trans*-2-hexenedioic acid (Fig. 3b). The results indirectly demonstrated expression of *paaJ, paaH, paaF*, and *tesB* genes despite the operon organization with placing *paaF* and *tesB* at the downstream of each operon (Fig. 1). Downstream genes are known to be expressed weaker than the upstream genes in operon configurations [57]. However, the results also suggested that either expression of operons was not optimal for downstream genes or there was a bottleneck in the pathway.

### Implementation of full-length reverse β-oxidation in *C. glutamicum*

**Fig. 4:**
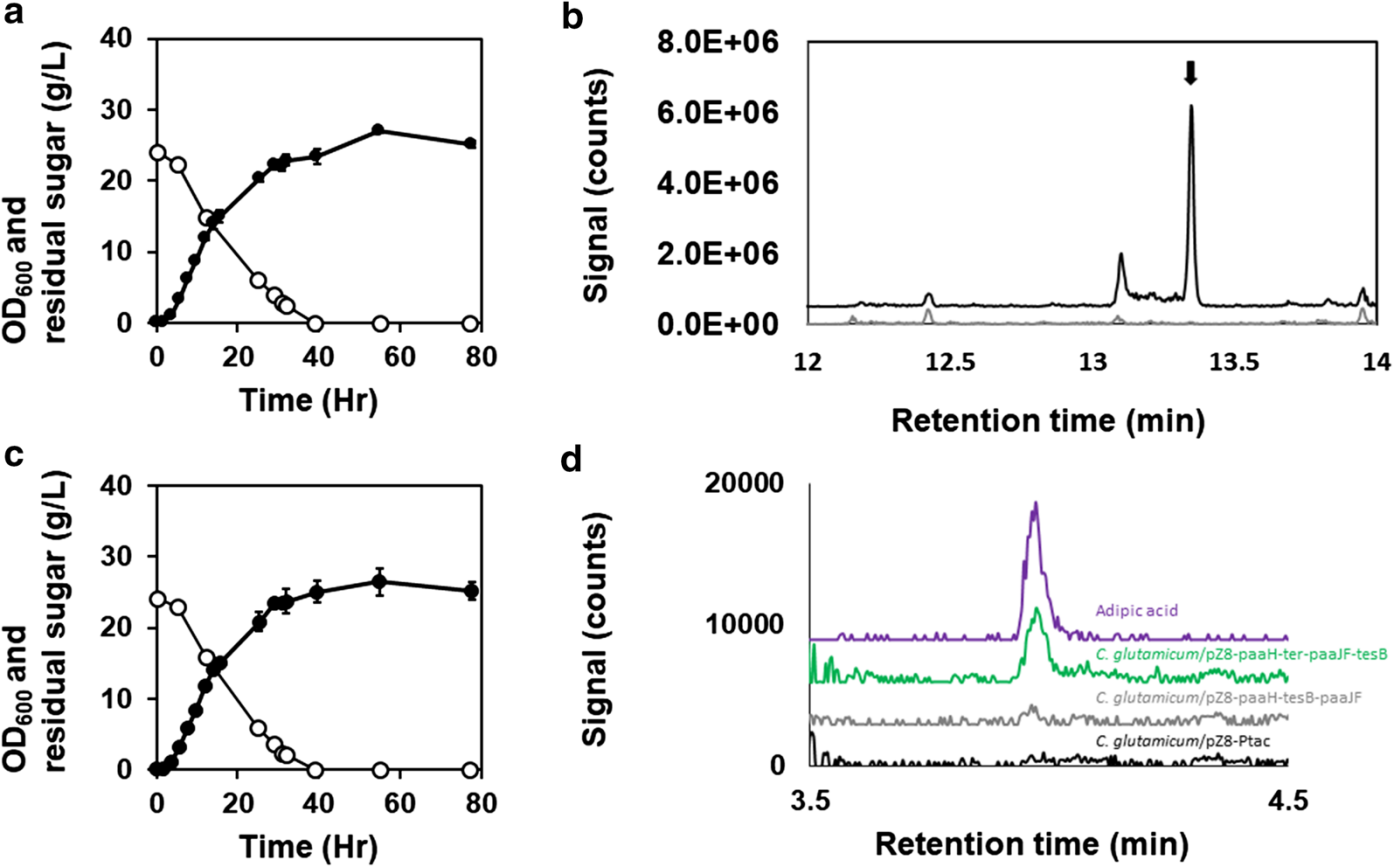
Shake-flask cultivation profile of *C. glutamicum* harboring pZ8-paaH-ter-paaJ-paaF-tesB. **a** Cultivation parameters include OD_600_ (filled circles) and residual sugar (empty circles). N = 3; error bars = standard deviation. **b** Ion (*m/z* = 363) extracted chromatogram obtained by GC/MS of the cultivation broth (t = 55 h) of *C. glutamicum* harboring pZ8-paaH-ter-paaJ-paaF-tesB (black line) and an empty vector (grey line). The peak corresponding to 3-hydroxyadipate is indicated with a black arrow. **c** Cultivation profile of *C. glutamicum* harboring pZ8-Ptac showing OD_600_ (filled circles) and residual sugar (empty circles). N = 3; error bars = standard deviation. (**d**) Ion (*m/z* = 145.0506) extracted chromatogram obtained by LC/MS of adipic acid standard (purple), cultivation broth of *C. glutamicum* harboring pZ8-paaH-ter-paaJ-paaF-tesB (green), pZ8-paaH-tesB-paaJF (grey), and an empty vector (black)

Addition of Ter from *T. denticola* [48] completed the reverse β-oxidation pathway for adipic acid production in *C. glutamicum* (Fig. 1). Overexpression of *ter* from *T. denticola* (KEGG ID: TDE0597) was shown to reduce *trans*-2-enoyl-CoA in engineered *E. coli* [16]. To express codon-optimized *paaJ*, *paaH*, *paaF*, *ter*, and *tesB* on a single vector, the genes were reorganized in two separate operons, each driven by a *tac* promoter and constructed in such a way that *paaF* was expressed after *paaJ* and *ter* after *paaH.* Expression of *tesB* was again given the lowest priority and was placed in the last position of the second operon, downstream of *paaF*. Translation of *paaF*, *ter*, and *tesB* was enabled by each accompanying synthetic RBS. *C. glutamicum* transformed with the resulting pZ8-paaH-ter-paaJ-paaF-tesB plasmid reached OD_600_ of 25.2 after 78 h of flask-batch cultivation (Fig. 4a). MSTFA derivatization of the cultivation broth followed by GC/MS revealed the presence of 3-hydroxyadipate (Fig. 4b, c) but not *trans*-2-hexenedioic acid or adipic acid. However, concentrating cultivation broth 5-fold followed by LC-MS revealed presence of 37.3 µg/L of adipic acid (Fig. 4d). Analysis of LC-MS/MS further confirmed the identity of adipic acid from the cultivation broth (Additional file 5: Fig. S3). The MS/MS spectra obtained was also in agreement with publicly available database entry (HMDB0000448) [58]. *C. glutamicum* harboring an empty vector also reached OD_600_ of 25.2 (Fig. 4c), and no intermediate compounds were detected in the control strain (Fig. 4b-d). The presence of an intermediate and adipic acid confirmed that even when *tesB* expression was given the least priority, release of CoA was not hindered by the pZ8-paaH-ter-paaJ-paaF-tesB construct. A possible reason for this is that native thioesterase (i.e., NCgl1600) activity was strong enough to release CoA from 3-hydroxyadipyl-CoA or that expression and functionality of *tesB* were achieved. However, these results also pointed to the need for a new strategy to assess and improve gene expression of the attempted synthetic pathway.

## Discussion

In this study, we aimed to investigate the reverse β-oxidation pathway and its functionality in engineered *C*. *glutamicum*. In the final strain, detection of 3-hydroxyadipic acid as major byproduct, small amount of adipic acid, and concomitant absence of *trans*-2-hexenedioic acid pointed to inefficient PaaF activity, although Ter functionality should be assessed separately as well (Figs. 3, 4). Such pattern indicated that the dehydration of 3-hydroxyadipyl-CoA was the bottleneck step. The *paaF* and *ter* genes were each placed downstream in the respective operons (Fig. 1), since placing the upstream pathway genes in the upstream of operon organization has proven advantageous [34, 35]. This also might have led to decreased expression of *paaF*. Hence, a possible strategy to overcome the expression challenge is to switch the operon organization or to place additional promoters for improved *paaF* and *ter* expression [57]. A potential strategy to overcome dehydration step is to use another enzyme, such as Ech (enoyl-CoA hydratase) from *Ralstonia eutropha*, Crt (crotonase) from *Clostridium acetobutylicum* [14], or Tfu_0067 from *T. fusca* [15], as it might be that *E. coli* PaaF is simply less compatible with *C. glutamicum* for some unknown reason.

Lack of 3-oxoadipic acid in the cultivation broth can be potentially explained by the presence of PcaF, which is encoded by *cg2625* (EC:2.3.1.9) and involved in degradation of 3-oxoadipic acid [59]. Although *paaJ* overexpression might have allowed an equilibrium between 3-oxoadipyl-CoA, acetyl-CoA, and succinyl-CoA, PcaF could have represented a potential source of disturbance by reversing the direction of the chemical reaction. We speculate that PcaF acts against PaaJ to actively degrade 3-oxoadipyl-CoA. Furthermore, because 3-oxoadipic acid synthesized in the presence of PaaJ and TesB can be reverted to 3-oxoadipyl-CoA by PcaIJ [59], chromosomal deletion of *pcaIJ* and *pcaF* appears necessary to prevent degradation of 3-oxoadipyl-CoA and 3-oxoadipic acid.

Once bottleneck step of the dehydration of 3-hydroxyadipyl-CoA is overcome, further strategies to improve adipic acid production might include expression optimization by engineering the reductive branch of TCA cycle [60], RBS switching [61], alternating reducing equivalents between NADH^+^ and NADPH [62], media engineering [63], eliminating competing pathways [64], using a different thioesterase for CoA removal [24], and removing any pathways leading to the formation of byproducts [65]. A switch in reducing equivalent can be achieved using *trans*-2-enoyl-CoA reductase (E.C. 1.3.1.38), which accepts NADPH, an abundant cofactor in *C. glutamicum*. Competing reaction can be eliminated by deleting *pcaF*, because the reverse degradation flux must be engineered to overcome the catabolic step [66]. Possible strain and pathway engineering strategies to improve titer and productivity have been discussed previously [67–69].

*C. glutamicum* offers various benefits, such as elevated tolerance to acids, strong TCA flux, and natural excretion of acetic and succinic acid upon metabolic stress. Its use of acetyl-CoA and succinyl-CoA in the initial steps was hypothesized to favor adipic acid production. However, while condensation of acetyl-CoA and succinyl-CoA was indeed successful, directing the flux toward adipic acid proved either impossible or insufficient. Hence, several challenges need to be overcome when engineering *C. glutamicum* for adipic acid production.

### Conclusions

The present study demonstrated the feasibility of the synthetic reverse β-oxidation pathway by showcasing adipic acid biosynthesis in *C. glutamicum*. This work laid the groundwork for future development of *C. glutamicum* as a microbial factory for adipic acid production. For the subsequent studies, metabolic flux should be engineered to overcome the present bottleneck represented by dehydration of 3-hydroxyadipyl-CoA, while chromosomal deletion of catabolic genes will bring further advantages.

## Methods

### Strains and plasmids

Primers (Additional file 6: Table S3) used in this study were synthesized at Eurofins Genomics (Ebersberg, Germany), Thermo Fisher (Waltham, MA, USA) or Sigma-Aldrich (St. Louis, MO, USA). Codon-optimization for *C*. *glutamicum* (Additional file 2: Table S2) and subsequent gene synthesis were carried out at GenScript (Piscataway, NJ, USA). All constructed vectors were sequence-verified at Eurofins Genomics or Macrogen Europe (Amsterdam, the Netherlands). Plasmid pZ8-P*tac* (Addgene plasmid number 74,064) was a gift from Timothy Lu (MIT, Cambridge, MA, USA) [54]. General PCR conditions were as follows: 95 °C for 5 min; 28 cycles at 95 °C for 30 s, 55 °C for 30 s, 72 °C for 1 min; and a final extension at 72 °C for 7 min. The PCR was carried out with Phusion High-Fidelity DNA Polymerase (Thermo Fisher) in a total reaction volume of 50 µL. All restriction enzymes were purchased from Thermo Fisher. *E*. *coli* NEB^®^5-alpha and NEB^®^10-beta from New England Biolabs (Ipswich, MA, USA) were used for general cloning purposes. The *C*. *glutamicum* ATCC 13,032 strain used in this study is a lab stock. Chemicals used in this study were purchased from Merck (Kenilworth, NJ, USA) or Thermo Fisher.

pZ8**-**paaJ**-**tesB was constructed by cloning codon-optimized *paaJ* and *tesB* into the pZ8-P*tac* vector. Primers 134_F_*Eco*RI and 133_i3R_*Kpn*I v2 were used to amplify *paaJ* and the product was digested with *Eco*RI/*Kpn*I. Primers tesB_F_*Kpn*I and tesB_R_*BamH*I were used to amplify *tesB* and the product was digested with *Kpn*I/*Bam*HI. pZ8-P*tac* was digested with *Eco*RI/*Bam*HI and subsequently three-way ligated with the two PCR products. A 16-bp RBS (tttcacacaggaaaca) from the synthetic *lacUV5* promoter was placed between *paaJ* and *tesB* for *tesB* expression during this step.

Construction of pZ8**-**HB was carried out by replacing *ter* from pZ8**-**Hr with *tesB*. Codon-optimized *tesB* was PCR-amplified with primers tesB_F_*Kpn*I and tesB_R_*Bam*HI, and then digested with *Kpn*I/*Bam*HI. This fragment was ligated into pZ8-Hr previously digested with *Kpn*I/*Bam*HI to replace *ter*. An extra promoter and *paaJ* were amplified by primers PA146F_Gib and PA146R_Gib using pZ8**-**paaJ as template, and then inserted into pZ8**-**HB by Gibson assembly to yield pZ8-paaH-tesB-paaJ.

*Trans*-2-Hexenedioic acid producing vector, pZ8-paaH-tesB-paaJF, was created by adding an extra promoter, *paaJ*, and *paaF* fragments to already prepared pZ8-HB. The promoter, paaJ, and paaF was PCR amplified using the primers, 143_F_Gibson and 143_R_Gib, and introduced into *Bam*HI digested pZ8-HB.

Vector pZ8**-**paaH**-**ter**-**paaJ**-**paaF**-**tesB was constructed in a stepwise manner. A codon-optimized version of *E. coli paaH* was PCR-amplified using primers 133_i1F_EcoRI and 133_i1R_*Kpn*I (Additional file 6: Table S3), and then digested with *Eco*RI and *Kpn*I. A codon-optimized version of *T. denticola ter* was PCR-amplified using primers 133_i2F_*Kpn*I and 133_i2R_*Bam*HI and digested with *Kpn*I and *Bam*HI. The digested PCR products were three-way ligated with *Eco*RI/*Bam*HI-digested pZ8-P*tac* to yield pZ8**_**Hr. A 16-bp untranslated region (UTR) sequence (tttcacacaggaaaca) [55] including an RBS was introduced between the two genes for codon-optimized *ter* expression. To construct pZ8_HrJF, an extra *tac* promoter, codon-optimized *paaJ*, and *paaF* were introduced into pZ8**_**Hr. The codon-optimized version of *paaJ* was PCR-amplified using primers pre132_i3F_*Bam*HI and 133_i3R_*Kpn*I v2, and subsequently digested with *Bam*HI/*Kpn*I. The codon-optimized *paaF* fragment (PCR-amplified with primers 133_i4F_*Kpn*I and 133_i4R_*Pst*I v2) was digested with *Kpn*I/*Pst*I. Digested *paaJ* and *paaF* fragments were subsequently three-way ligated into pZ8_Hr previously digested with *Bam*HI/*Pst*I. As with pZ8_Hr, an additional 16-bp UTR (tttcacacaggaaaca) with RBS was introduced at this point to allow for *paaF* expression. Thus, an extra promoter was PCR-amplified with primers tac_BamHI_F and tac_BamHI_R, digested with *Bam*HI, and then ligated upstream of *paaJ* at the *Bam*HI site. The resulting plasmid was named pZ8_HrJF. For pZ8**-**paaH**-**ter**-**paaJ**-**paaF**-**tesB construction, the *tesB* gene was added to pZ8_HrJF. Codon-optimized *tesB* was PCR-amplified using primers 133_i5F_PstI and 133_i5R_PstI, and then digested with *Pst*I. The resulting fragment was ligated into pZ8_HrJF at the *Pst*I site downstream of *paaF*.

pZ8**-**paaJ was constructed by removing *tesB* from pZ8**-**paaJ**-**tesB. This was achieved by digesting pZ8**-**paaJ**-**tesB with *Kpn*I and *Bam*HI, followed by ligation, whereby a short linker was placed at the 3′ end of *paaJ* as *Kpn*I and *Bam*HI were not compatible. The linker consisted of oligos linker_sense and linker_antisense (Additional file 6: Table S3). This step introduced also an *Xba*I site between *Kpn*I and *Bam*HI for potential future usage.

### Pathway intermediate standards

Standards of pure 3-oxoadipic acid (O856825) and 3-hydroxyadipic acid (H943100) were purchased from Toronto research chemicals (North York, ON, Canada). *Trans*-2-hexenedioic acid was synthesized by our group during a previous study [70].

### Media

Pre-culture medium included (per liter): 40 g brain heart infusion, 10 g glucose, and 20 g D-sorbitol. Flask cultivation medium included (per liter): 25 g glucose, 2 g MgSO_4_·7H_2_O, 1 g K_2_HPO_4_, 1 g KH_2_PO_4_, 1 g urea, 20 g (NH_4_)_2_SO_4_, 10 g yeast extract, 0.65 mg (NH_4_)_6_Mo_7_O_24_, 7.726 mg CaCl_2_·2H_2_O, 11.35 µg biotin, 4.57 mg thiamin, 9 mg FeSO_4_·7H_2_O, 7.88 mg MnSO_4_·H_2_O, 5.5 mg CuSO_4_· 5H_2_O, 10.06 mg ZnSO_4_·7H_2_O, and 1.88 mg NiCl_2_·6H_2_O. Additionally, 20 g/L of CaCO_3_ was added to each flask as buffer and CaCO_3_ was removed by diluted HCl post-cultivation.

### Cultivation conditions

Cultivation was carried out in 100-mL Erlenmeyer flasks at 30 °C and 200 rpm in a shaking incubator (KS 4000 I control; IKA-Werke GmbH, Staufen im Breisgau, Germany). Each strain was maintained in 60 % glycerol solution and was inoculated in 5 mL pre-culture medium. After 17–19 h, when OD_600_ reached 5.5–6.0, 1 mL of pre-culture was spun down at 3000 rcf and the pellet was resuspended in 25 mL of cultivation medium. When applicable, isopropyl β-d-1-thiogalactopyranoside at a final concentration of 1 mM was added at OD_600_ 0.5–0.8. Neomycin at a final concentration of 50 µg/L was added as a selective marker during each cultivation.

### Cell growth and routine metabolite analysis

Cell growth during cultivation was monitored by measuring OD_600_ on a Genesys 20 spectrophotometer (Thermo Fisher).

The concentration of glucose and organic acids in cultivation broth was analyzed by a Jasco (Tokyo, Japan) LC-4000 high-performance liquid chromatography system equipped with an autosampler (AS-4150), a pump (PU-4180), a column oven (CO-4061), a UV detector (RI-4030), and an RI detector (UV-4075). The mobile phase consisted of 5 mM H_2_SO_4_ and was pumped at constant flow rate of 0.8 mL/min. A Rezex ROA-organic acid H + column (Phenomenex, Torrance, CA, USA) maintained at 80 °C was used to separate the metabolites in the cultivation broth. The concentration of each compound was determined by Jasco ChomNAV software (version 2.03.03).

### Confirmation of 3-hydroxyadipic acid by GC/MS analysis

3-hydroxyadipic acid was identified by GC/MS as reported previously with minor modifications [19, 71]. Briefly, 40 µL of cell-free cultivation broth was lyophilized, resuspended in 50 µL pyridine, and reacted with 50 µL MeOX (20 g/L in pyridine) for 120 min at 30 °C. The metabolites were then derivatized with 50 µL MSTFA at 60 °C for 1 h prior to injection.

The GC/MS system was equipped with a Focus GC (Thermo Fisher), ISQ MS, and PAL COMBI-xt autosampler. A Zebron ZB-5MS column (Phenomenex) was used, with 1.1 mL/min gas flow and helium as the carrier gas. The column temperature gradient was as follows: initial hold at 60 °C for 1 min; ramp at 10 °C/min to 325 °C; and final hold at 325 °C for 10 min. Equilibration time was 0.5 min and preparatory run time out was 10 min. The inlet temperature was 250 °C. The electron multiplier was operated at 70 eV with scanning acquisition mode. In total, 1 µL of sample was injected for each analysis with split-less mode. The ion (MS) source was set at 250 °C, mass filter (MS Quad) at 150 °C, and MS transfer line at 290 °C.

### Solid-phase extraction

For sample clean up, 33 μm polymeric reverse-phased solid-phase extraction (SPE) column (200 mg/3 mL; 8B-S100-FBJ) was used with minor modification from manufacturer’s protocols (Phenomenex). The precondition and equilibration steps were according to the manufacturer’s protocols. The 5 mL of cell-free cultivation broth was pretreated with final 1 % of formic acid prior to loading to the column. The column was washed with 1 mL water and eluted with 1 mL methanol. Methanol was removed by evaporation prior to next steps.

### Detection of *trans*-2-hexenedioic acid and adipic acid by LC-MS

For detection of adipic acid and *trans*-2-hexenedioic acid, SPE treated samples were analyzed using LC-MS. A Dionex Ultimate 3000 RS UPLC with a diode array detector (DAD) was coupled to a Bruker Maxis QTOF MS with electrospray ionization (ESI) (Bruker Daltonics, Bremen, Germany). The system was equipped with a reversed phase column (Kinetex; 1.7 μm, F5, 100 Å, 150 mm × 2.1 mm; Phenomenex) and was maintained at 40 °C. Gradient elution was utilized for separation and a constant flow rate of 400 µL/min was held throughout the run. Two eluents were used in the gradient elution: eluent A (H2O, 20 mM formic acid) and eluent B (ACN, 20 mM formic acid). The gradient started with 10 % eluent B rising to 70 % over 6.7 min, followed by 70–100 % eluent B over 1.3 min, then held at 100 % eluent B for 3 min. Analysis was carried out in negative ESI. The scan range was *m/z* 75–1250 with 2 spectra per second. For the targeted fragmentation a collision energy of 15 eV was used.

Prior to the analysis of samples, the QTOF-MS was calibrated using a sodium formate calibrant. In addition, all data files were recalibrated with an internal standard of sodium formate injected prior to initial sample elution for each sample.

## Supplementary Information

**Additional file 1: Table S1**. Strains and plasmids used in this study.

**Additional file 2: Table S2**. Sequences of codon-optimized genes.

**Additional file 3: Figure S1. **GC/MS analysis of authentic 3-oxoadipic acid standard. **(a)** Ion (*m/z* = 318) extracted chromatogram and **(b)** *m/z* fragmentation pattern for MeOX and MSTFA-derivatized 3-oxoadipic acid obtained by GC/MS. The inset in (b) corresponds to the GoLM metabolome database [72] entry (A166019) for the same compound. (**c**) Comparison of fragmentation pattern of 3-oxoadipic acid standard (upper) and database entry (lower).

**Additional file 4: Figure S2. **GC/MS analysis of authentic 3-hydroxyadipic acid standard.** (a)** Ion-extracted (*m/z* = 363) chromatogram of MSTFA-derivatized 3-hydroxyadipic acid standard. **(b)** *m/z* fragmentation spectrum of 3-hydroxyadipate standard. (**c**) The NIST database entry (79677) for the same compound.

**Additional file 5: Figure S3. **LC-MS/MS analysis of adipic acid in the cultivation broth. (**a**) Extracted ion chromatogram (*m/z* 145.05) of cultivation broth of *C. glutamicum* harboring pZ8-paaH-ter-paaJ-paaF-tesB (green), pZ8- paaH-tesB-paaJF (grey), and an empty vector (black). Adipic acid standard is shown for comparison of retention time (purple). (**b**) MS/MS of *m/z* 145.05 of precursor ion ([M-H]^−^) from engineered *C. glutamicum* (green) and adipic acid standard (purple).

**Additional file 6: Table S3.**Primers used in this study. Restriction enzyme sites are indicated in boldface. Extra RBS sequences for expression of the immediate downstream gene are underlined.

## Data Availability

All data generated or analyzed during this study are included in this published article [and its supplementary information files].
