# Supplementary material for: Exploring functionality of the reverse β-oxidation pathway in Corynebacterium glutamicum for production of adipic acid
Source: Microb Cell Fact. 2021 Aug 4;20:155. doi: 10.1186/s12934-021-01647-7 (PMC8336102; doi:10.1186/s12934-021-01647-7)
Supplement: Supplementary file 1 — Additional file 1: Table S1. Strains and plasmids used in this study. [file 12934_2021_1647_MOESM1_ESM.docx]

**Additional file 1: Table S1** strains and plasmids used in this study

| **Strains** | Remarks | Reference or source |
| --- | --- | --- |
| *C. glutamicum* | Wild type strain (DSM 20300 / ATCC 13032) | Lab stock |
| *E. coli* NEB 5 alpha | General cloning purpose. Genotype: *fhuA2 Δ(argF-lacZ)U169 phoA glnV44 Φ80 Δ(lacZ)M15 gyrA96 recA1 relA1 endA1 thi-1 hsdR17* | New England Biolabs |
| *E. coli* NEB 10 beta | General cloning purpose. Genotype: Δ*(ara-leu) 7697 araD139  fhuA*Δ*lacX74 galK16 galE15 e14-*ϕ*80*d*lacZ*Δ*M15  recA1 relA1 endA1 nupG  rpsL*(Str^R^)*rph spoT1*Δ*(mrr-hsdRMS-mcrBC)* | New England Biolabs |
| **Plasmids** |  |  |
| pZ8-P*tac* | *E*. *coli* – *C*. *glutamicum* shuttle vector, *Km^R^*, P_tac_, *lacI^Q^* | [51] |
| pZ8_paaJtesB | pZ8-Ptac derivative, codon-optimized *paaJ*, codon-optimized *tesB* | This study |
| pZ-HB | pZ8-Ptac derivative, codon-optimized *paaH*, codon-optimized *tesB* | This study |
| pZ8_paaH_tesB-paaJ | pZ8-Ptac derivative, codon-optimized *paaH*, codon-optimized *tesB*, codon-optimized *paaJ* | This study |
| pZ8-paaH-tesB-paaJF | pZ8-Ptac derivative, codon-optimized *paaH*, codon-optimized *tesB*, codon-optimized *paaJ,* codon-optimized *paaF* | This study |
| pZ8_paaH-ter-paaJ-paaF-tesB | pZ8-Ptac derivative, codon-optimized *paaH*, codon-optimized *ter,* codon-optimized *paaJ*, codon-optimized *paaF*, codon-optimized *tesB* | This study |
| pZ8_Hr | pZ8-Ptac derivative, codon-optimized *paaH*, codon-optimized *ter* | This study |
| pZ8_HrJF | pZ8-Ptac derivative, codon-optimized *paaH*, codon-optimized *ter,* codon-optimized *paaJ*, codon-optimized *paaF* | This study |
| pZ8_paaJ | pZ8-Ptac derivative, codon-optimized *paaJ* | This study |
