# Supplementary material for: Exploring functionality of the reverse β-oxidation pathway in Corynebacterium glutamicum for production of adipic acid
Source: Microb Cell Fact. 2021 Aug 4;20:155. doi: 10.1186/s12934-021-01647-7 (PMC8336102; doi:10.1186/s12934-021-01647-7)
Supplement: Supplementary file 2 — Additional file 2: Table S2. Sequences of codon-optimized genes. [file 12934_2021_1647_MOESM2_ESM.docx]

**Additional file 2: Table S2. Sequences of the codon optimized genes**

| paaH | ATGATGATCAACGTGCAGACCGTGGCAGTTATCGGCTCCGGCACCATGGGTGCAGGTATCGCCGAAGTGGCAGCATCCCACGGTCACCAGGTCCTGCTCTACGATATCTCCGCCGAGGCTCTGACCCGCGCAATCGATGGCATCCACGCACGTCTCAACTCCCGTGTGACCCGTGGCAAGCTGACCGCAGAAACCTGCGAGCGCACCCTGAAGCGCCTCATCCCAGTCACCGATATCCACGCCCTGGCAGCAGCAGATCTCGTGATCGAAGCTGCATCCGAACGCCTGGAAGTGAAGAAGGCACTGTTCGCACAGCTCGCCGAGGTGTGCCCACCACAGACCCTGCTCACCACCAACACCTCTTCCATCTCCATCACCGCCATCGCCGCTGAAATCAAGAACCCAGAGCGCGTCGCTGGCCTCCACTTCTTCAACCCAGCACCAGTCATGAAGCTCGTGGAAGTGGTCTCCGGTCTGGCTACCGCAGCCGAAGTGGTCGAGCAGCTGTGCGAGCTGACCCTCTCCTGGGGCAAGCAGCCAGTGCGTTGCCACTCCACCCCAGGCTTCATCGTCAACCGCGTGGCTCGCCCATACTACTCCGAAGCTTGGCGCGCACTCGAAGAGCAGGTGGCTGCACCAGAGGTCATCGATGCTGCTTTGCGCGATGGTGCAGGTTTCCCAATGGGTCCACTGGAACTCACCGATCTGATCGGCCAGGACGTCAACTTCGCCGTGACCTGCTCCGTCTTCAACGCTTTCTGGCAGGAACGCCGCTTCCTGCCATCCCTCGTGCAGCAGGAGCTGGTCATCGGCGGCCGCCTCGGCAAGAAGTCCGGTCTGGGCGTGTACGATTGGCGCGCTGAACGTGAGGCAGTGGTGGGTCTGGAAGCTGTGTCCGATTCCTTCTCCCCAATGAAGGTCGAAAAGAAGTCCGACGGCGTGACCGAGATCGATGACGTCCTGCTCATCGAGACCCAGGGTGAAACCGCACAGGCACTGGCTATCCGTCTGGCTCGCCCAGTGGTCGTGATCGATAAGATGGCAGGCAAGGTCGTGACCATCGCAGCAGCAGCAGTGAACCCAGACTCCGCCACCCGCAAGGCTATCTACTACTTGCAGCAGCAGGGCAAGACCGTGCTCCAGATCGCCGATTACCCAGGCATGCTCATCTGGCGCACCGTCGCTATGATCATCAACGAAGCCCTGGACGCTCTCCAGAAGGGCGTGGCATCCGAGCAGGATATCGACACCGCCATGCGCCTGGGCGTCAACTACCCATACGGTCCACTGGCATGGGGCGCCCAGCTGGGCTGGCAGCGCATCCTCCGCCTGCTCGAAAACTTGCAGCACCACTACGGCGAAGAGCGCTACCGCCCATGCTCCTTGCTGCGTCAGCGTGCACTGCTGGAATCCGGCTACGAGTCCTAA |
| --- | --- |
| Ter | ATGATCGTGAAGCCAATGGTCCGCAACAACATCTGCCTCAACGCTCACCCACAGGGCTGCAAGAAGGGCGTGGAGGATCAGATCGAATACACCAAGAAGCGCATCACCGCAGAAGTCAAGGCAGGCGCCAAGGCTCCAAAGAACGTGCTGGTGCTGGGCTGCTCCAACGGTTACGGTCTGGCATCCCGCATCACCGCAGCCTTCGGTTACGGTGCTGCAACCATCGGCGTGTCCTTCGAGAAGGCCGGCTCCGAAACCAAGTACGGCACCCCAGGCTGGTACAACAACCTGGCTTTCGACGAGGCCGCTAAGCGCGAAGGCCTCTACTCCGTGACCATCGATGGCGACGCATTCTCCGATGAGATCAAGGCCCAGGTCATCGAAGAAGCAAAGAAGAAGGGCATCAAGTTCGACCTGATCGTGTACTCCCTGGCATCCCCAGTCCGTACCGATCCAGACACCGGCATCATGCACAAGTCCGTGCTCAAGCCATTCGGCAAGACCTTCACCGGCAAGACCGTCGATCCATTCACCGGCGAGCTGAAGGAAATCTCCGCCGAACCAGCTAACGATGAAGAGGCAGCCGCTACCGTGAAGGTCATGGGCGGCGAGGACTGGGAACGCTGGATCAAGCAGCTGTCCAAGGAAGGCCTGCTCGAAGAGGGCTGCATCACCCTCGCATACTCCTACATCGGCCCAGAAGCAACCCAGGCCCTGTACCGCAAGGGCACCATCGGCAAGGCAAAGGAGCACCTGGAAGCAACCGCCCACCGCCTCAACAAGGAAAACCCATCCATCCGCGCCTTCGTGTCCGTCAACAAGGGCCTCGTGACCCGCGCTTCCGCAGTGATCCCAGTCATCCCACTGTACCTCGCCTCCCTGTTCAAGGTCATGAAGGAGAAGGGCAACCACGAGGGCTGCATCGAACAGATCACCCGCCTGTACGCTGAACGCCTCTACCGCAAGGATGGCACCATCCCAGTGGACGAAGAGAACCGCATCCGCATCGATGACTGGGAGCTGGAAGAGGATGTGCAGAAGGCTGTCTCCGCACTCATGGAAAAGGTGACCGGCGAGAACGCAGAATCCTTGACCGATCTGGCTGGCTACCGCCACGACTTCCTGGCTTCCAACGGCTTCGATGTGGAAGGCATCAACTACGAAGCAGAGGTCGAACGCTTCGACCGCATCTAA |
| paaJ | ATGCGCGAAGCCTTCATCTGCGATGGCATCCGCACCCCAATCGGCCGCTACGGCGGCGCTCTGTCCTCCGTGCGCGCAGATGACCTCGCAGCCATCCCACTGCGCGAACTGCTCGTGCGCAACCCACGCCTCGATGCAGAGTGCATCGATGACGTCATCCTGGGCTGCGCTAACCAGGCAGGTGAAGACAACCGTAACGTGGCACGTATGGCTACCTTGCTGGCTGGTCTGCCACAGTCCGTCTCCGGCACCACCATCAACCGCCTGTGCGGCTCCGGTCTGGATGCACTGGGTTTCGCTGCACGCGCAATCAAGGCTGGTGATGGTGACCTGCTCATCGCAGGCGGCGTGGAATCCATGTCCCGCGCACCATTCGTCATGGGCAAGGCAGCTTCCGCATTCTCCCGCCAGGCTGAGATGTTCGATACCACCATCGGCTGGCGCTTCGTGAACCCACTCATGGCCCAGCAGTTCGGCACCGACTCCATGCCAGAAACCGCAGAGAACGTCGCCGAACTGCTCAAGATCTCCCGCGAGGATCAGGACTCCTTCGCACTGCGTTCCCAGCAGCGTACCGCAAAGGCTCAGTCCTCCGGCATCCTCGCCGAAGAGATCGTGCCAGTGGTCCTGAAGAACAAGAAGGGCGTGGTCACCGAAATCCAGCACGATGAGCACCTGCGCCCAGAAACCACCTTGGAGCAGCTGCGCGGCCTGAAGGCTCCATTCCGTGCAAACGGCGTGATCACCGCTGGCAACGCATCCGGCGTCAACGATGGTGCAGCCGCTCTCATCATCGCCTCCGAACAGATGGCAGCCGCTCAGGGTCTGACCCCACGCGCTCGTATCGTGGCAATGGCCACCGCTGGCGTCGAACCACGTCTCATGGGTCTGGGTCCAGTGCCAGCAACCCGTCGCGTCCTGGAGCGTGCTGGTCTGTCCATCCACGATATGGACGTGATCGAACTGAACGAGGCATTCGCAGCACAGGCACTCGGCGTGCTGCGTGAACTGGGCCTCCCAGATGACGCAC CACACGTCAACCCAAACGGCGGCGCAATCGCACTGGGTCACCCAC TGGGCATGTCCGGTGCACGCCTGGCTCTCGCTGCATCCCACGAAC TCCACCGTCGCAACGGCCGCTACGCTCTGTGCACCATGTGCATCG GCGTGGGCCAGGGCATCGCAATGATCCTGGAGCGCGTCTAA |
| paaF | ATGTCCGAACTGATCGTCTCCCGCCAGCAGCGCGTGTTGCTGTTGACCCTGAACCGTCCAGCAGCCCGTAACGCTCTGAACAACGCACTCCTGATGCAGCTCGTCAACGAACTGGAGGCAGCAGCAACCGATACCTCTATCTCCGTCTGCGTGATCACCGGCAACGCTCGCTTCTTCGCAGCAGGCGCTGACCTGAACGAAATGGCAGAGAAGGATCTCGCCGCTACCCTGAACGACACCCGTCCACAGCTCTGGGCACGTCTCCAGGCTTTCAACAAGCCACTGATCGCAGCCGTGAACGGCTACGCACTGGGTGCTGGTTGCGAGCTGGCACTCCTGTGCGATGTGGTCGTTGCAGGTGAAAACGCACGTTTCGGTCTCCCAGAGATCACCCTGGGCATCATGCCAGGTGCAGGCGGCACCCAGCGTCTGATCCGCTCCGTCGGCAAGTCCCTCGCCTCCAAGATG GTGCTGTCCGGCGAATCCATCACCGCTCAGCAGGCACAGCAGGCTGGTCTGGTCTCCGATGTGTTCCCATCCGACCTCACCCTGGAGTACGCTCTCCAGCTGGCATCCAAGATGGCACGTCACTCCCCACTCGCACTCCAGGCTGCAAAGCAGGCTCTCCGCCAGTCCCAGGAAGTCGCTCTCCAGGCAGGTTTGGCACAGGAGCGTCAGCTGTTCACCCTCCTGGCAGCTACCGAAGATCGCCACGAGGGCATCTCCGCATTCCTCCAGAAGCGCACCCCAGACTTCAAGGGCCGCTAA |
| tesB | ATGAACACCCTCACCCAGGAACTGGTGGAGCTGCTCTCCCTCGAGAAGCTGGAAGAGAACCTCTACCGCGGCATGTCCCGCAACCTGGTGGGCAAGCGTGTCTTCGGTGGCCAGGTCCTGGGTCAGGCTTTGCGCGCAGCATCCTACACCACCGATCGTCCAGCACACTCCCTCCACGCCTACTTCCTGTACGGCGGCGACGTGAACGCACCAATCATCTACGAAGTCGATCCACTGCGCGACGGCAAGTCCTTCGTGTCCCGCCAGGTCCGTGCAATCCAGCACGGTCGCACCATCTTCTCCGCTATGGTGTCCTTCGCATCCCCAGAAGAGGGCCTCAACTACCAGAACGATATGCCAGACTACCCAGCACCAGAACAGCTGAAGTCCGAAGCCGAGCTGAAGCTCGGCCTGATCGATTTC GTGCCAGAGAACGTCCGCGCCTCCTTCATGCGCGAACGCCACATCGAGATCCGCCCAGTGGAACCAGTCAACCCATTCCAGCCACAGCCACAGGCTCCAACCAACGCACACTACATCCGCACCCACGATAAGATCGGCAAGGCCTTCGACCAGATCGCTCTCCACCAGTCCATCGTGGCTTTCTACTCCGACTTCACCCTCATGACCACCGCACTGAAGCCACACGGCCTCTCCTACCTGTCCCCATCCCTCCAGTGCGCCTCCATCGATCACACCATCTACTTCCACCGCCCACTCCGCGCTGACGAGTGGATGCTGTACGATATGGATGCAACCGTCTCCGCTGGCTCCCGCGGCCTGAACTTCGGTCGTATGTGGCAGAACGGCCTGCTCGTGTGCTCCACCGTCCAGGAAGGCCTCATGCGTCTGCGTGAAATCGAGACCCAGTAA |
