# Supplementary figures and images for: Exploring functionality of the reverse β-oxidation pathway in Corynebacterium glutamicum for production of adipic acid
Source: Microb Cell Fact. 2021 Aug 4;20:155. doi: 10.1186/s12934-021-01647-7 (PMC8336102; doi:10.1186/s12934-021-01647-7)

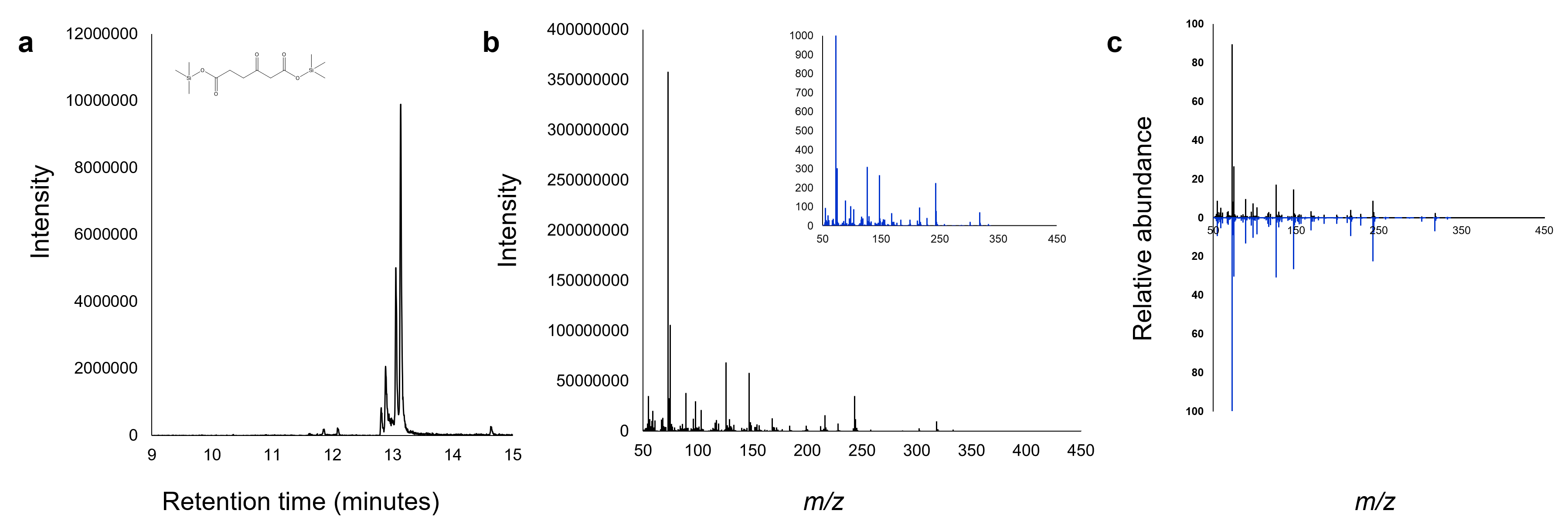

Supplement: Supplementary file 3 — Additional file 3: Figure S1. GC/MS analysis of authentic 3-oxoadipic acid standard. (a) Ion (m/z = 318) extracted chromatogram and (b) m/z fragmentation pattern for MeOX and MSTFA-derivatized 3-oxoadipic acid obtained by GC/MS. The inset in (b) corresponds to the GoLM metabolome database [72] entry (A166019) for the same compound. (c) Comparison of fragmentation pattern of 3-oxoadipic acid standard (upper) and database entry (lower). [file 12934_2021_1647_MOESM3_ESM.tif]

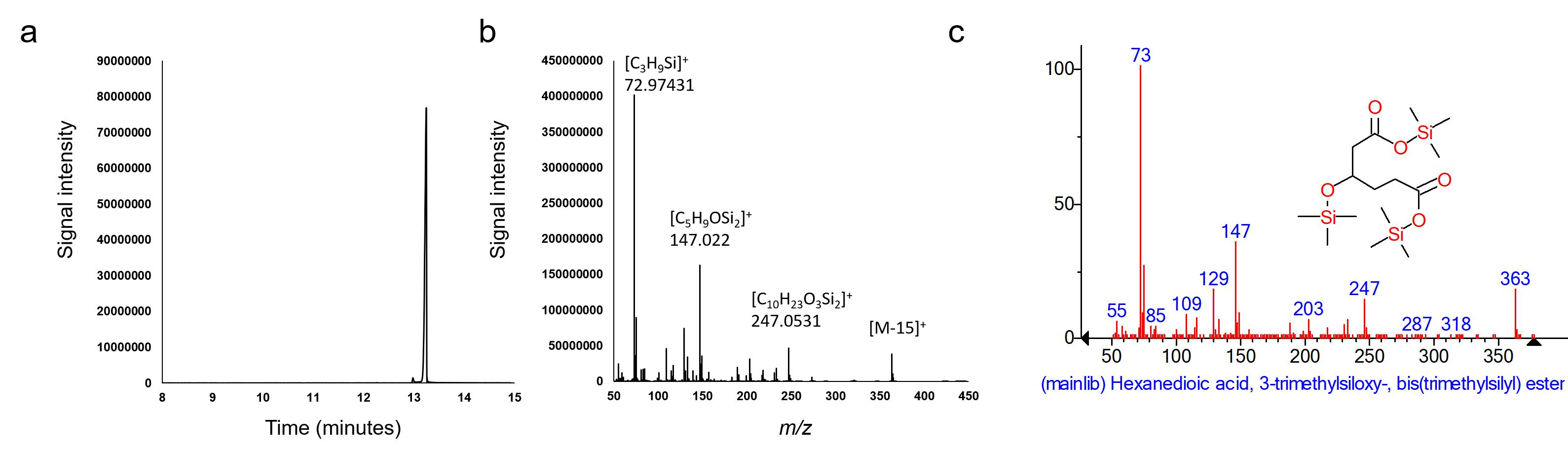

Supplement: Supplementary file 4 — Additional file 4: Figure S2. GC/MS analysis of authentic 3-hydroxyadipic acid standard. (a) Ion-extracted (m/z = 363) chromatogram of MSTFA-derivatized 3-hydroxyadipic acid standard. (b) m/z fragmentation spectrum of 3-hydroxyadipate standard. (c) The NIST database entry (79677) for the same compound. [file 12934_2021_1647_MOESM4_ESM.tif]

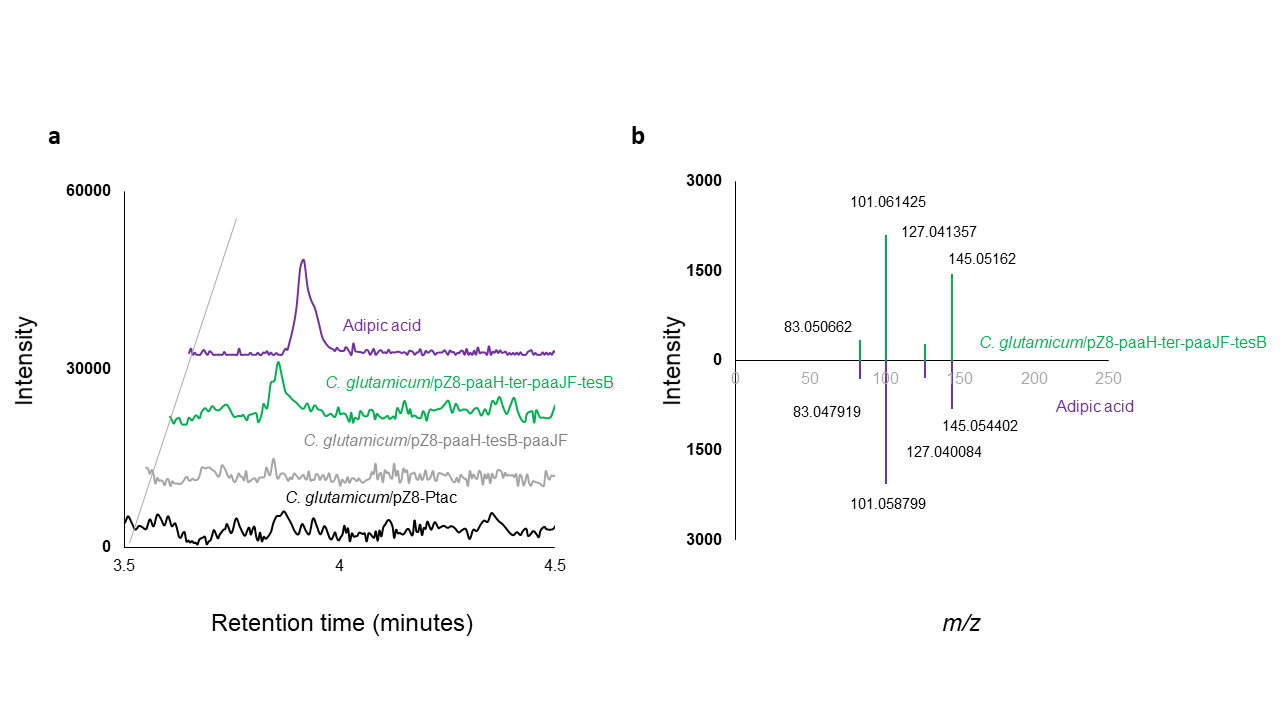

Supplement: Supplementary file 5 — Additional file 5: Figure S3. LC-MS/MS analysis of adipic acid in the cultivation broth. (a) Extracted ion chromatogram (m/z 145.05) of cultivation broth of C. glutamicum harboring pZ8-paaH-ter-paaJ-paaF-tesB (green), pZ8- paaH-tesB-paaJF (grey), and an empty vector (black). Adipic acid standard is shown for comparison of retention time (purple). (b) MS/MS of m/z 145.05 of precursor ion ([M-H]−) from engineered C. glutamicum (green) and adipic acid standard (purple). [file 12934_2021_1647_MOESM5_ESM.tif]
