# Supplementary material for: Exploring functionality of the reverse β-oxidation pathway in Corynebacterium glutamicum for production of adipic acid
Source: Microb Cell Fact. 2021 Aug 4;20:155. doi: 10.1186/s12934-021-01647-7 (PMC8336102; doi:10.1186/s12934-021-01647-7)
Supplement: Supplementary file 6 — Additional file 6: Table S3.Primers used in this study. Restriction enzyme sites are indicated in boldface. Extra RBS sequences for expression of the immediate downstream gene are underlined. [file 12934_2021_1647_MOESM6_ESM.docx]

**Additional file 6: Table S3** Primers used in this study. Restriction enzyme sites are indicated in boldface. Extra RBS sequences for expression of the immediate downstream gene are underlined.

| Name | Sequence (5’-3’) | Amplification target |
| --- | --- | --- |
| 134 F EcoRI | aaca**gaattc**ATGCGCGAAGCCTTCATCTG | *paaJ* |
| 133_i3R_KpnI v2 | gaaa**ggtacc**ttaGACGCGCTCCAGGATCA | *paaJ* |
| tesB_F_KpnI | ctaa**ggtacc**tttcacacaggaaacaATGAACACCCTCACCCAGGA | *tesB* |
| tesB_R_BamHI | cgac**ggatcc**ttaCTGGGTCTCGATTTCAC | *tesB* |
| PA146F_Gib | CCTCATGCGTCTGCGTGAAATCGAGACCCAGtaa**ggatcc**gctcaaggcgcactcccgtt | P*tac* |
| PA146R_Gib | catccgccaaaacagccaagcttggctgcagttaGACGCGCTCCAGGATCA | *paaJ* |
| 143 F Gibson | GGAAGGCCTCATGCGTCTGCGTGAAATCGAGACCCAGtaa**ggatcc**gctcaaggcgcactcccgtt | P*tac* |
| 143_R_Gib | tctcatccgccaaaacagccaagcttggctgcaggtcgac**ggatcc**ttaGCGGCCCTTGAAGTCTG | *paaF* |
| 133_i1F_EcoRI | aaca**gaattc**ATGATGATCAACGTGCAGAC | *paaH* |
| 133_i1R_KpnI | gaaa**ggtacc**ttaGGACTCGTAGCCGGATT | *paaH* |
| 133_i2F_KpnI | ctaa**ggtacc**tttcacacaggaaacaATGATCGTGAAGCCAATGGT | *ter* |
| 133_i2R_BamHI | gagc**ggatcc**ttaGATGCGGTCGAAGCGTT | *ter* |
| Pre132_i3F_*Bam*HI | Ctaa**ggatcc**ATGCGCGAAGCCTTCATCTG | *paaJ* |
| 133_i4F_KpnI | ctaa**ggtacc**tttcacacaggaaacaATGTCCGAACTGATCGTCTC | *paaF* |
| 133_i4R_PstI v2 | gaaa**ctgcag**ttaGCGGCCCTTGAAGTCTG | *paaF* |
| Tac_BamHI F | ctaa**ggatcc**gctcaaggcgcactcccgtt | P*tac* |
| Tac_BamHI R | GCAT**ggatcc**tgtttcctgtgtgaaattgt | P*tac* |
| 133_i5F_PstI | ctaa**ctgcag**tttcacacaggaaacaATGAACACCCTCACCCAGGA | *tesB* |
| 133_i5R_PstI | ttgg**ctgcag**ttaCTGGGTCTCGATTTCAC | *tesB* |
| Linker sense | cCATATGTCTAGAg | *paaJ* |
| Linker antisense | gatccTCTAGACATATGggtac | *paaJ* |
